# Lyophilized Probiotic Lactic Acid Bacteria Viability in Potato Chips and Its Impact on Oil Oxidation

**DOI:** 10.3390/foods9050586

**Published:** 2020-05-05

**Authors:** Heba Mostafa

**Affiliations:** Food Science Department, Faculty of Agriculture, Cairo University, Giza 12613, Egypt; Hebabiotech@agr.cu.edu.eg or Hebabiotech@gmail.com; Tel.: +02-010-022-108-22

**Keywords:** probiotic potato chips, lactic acid bacteria lyophilization, cryoprotectant, antioxidation

## Abstract

To produce a new probiotic-containing food product, potato chips, as the most preferred fast food, were chosen. Preferably, it should be preserved for a long period without oxidation. The presented study aimed to compare potato chips containing two lyophilized probiotic lactic acid bacteria (*Bifidobacterium longum* ATCC 15708 and *Lactobacillus helveticus* LH-B02) in order to retard lipid oxidation. Lyophilization of probiotics was carried out into two cryoprotective media—skim milk (SM) and gelatin/glycerol (GG) as lactose-free medium. Results revealed that GG and SM media were the most suitable for lyophilization of *B. longum* and *L. helveticus*, respectively. The lyophilized live cells were incorporated in potato chips, packed and their effect on oil oxidation was assessed. Results showed that the lyophilized *B. longum* in SM remained alive at 6.5 log CFU/g for 4 months at 30 °C. Interestingly, potato chip bags containing *B. longum* lyophilized in SM medium exhibited a decrease in peroxide value (PV) and acid value (AV) of the extracted oil by 40.13% and 25%, respectively, compared to the control bags. The created probiotic potato chips containing *B. longum* fulfill the criteria of the probiotic product besides the prime quality and sensory attributes.

## 1. Introduction

Today, with a busy lifestyle, individuals, especially young and adolescents, prefer to consume ‘ready-to-eat’ snack foods. The most popular and over-consumed product is potato chips, which are eaten as a snack food, side dish or appetizer. Potato chips may be consumed 3 times/week or more [1,2]. Potato chips are considered a starchy product (total carbohydrate in the range of 60–63.6%) in addition to fat (33–40%) and dietary fibers (1–1.6%) [3,4,5]. Chips also provide other important micronutrients such as sodium (480 mg/100 g) and potassium (166 mg/100 g) [3]. Potato also contains a variety of phytonutrients, most notably carotenoids and phenolic acids, mainly chlorogenic acid [6,7].

By deep oil frying, potato chips absorb a considerable quantity of oil. The final fat content ranges from 35% to 38% based on the total weight [8]. These high oil levels are not only important for nutritional quality but also have a dominant influence on the flavor, calories supplied, and their shelf life [9]. High surface-to-volume ratio of potato chips results in oxidative deterioration of the stored product. Also, lipid oxidation can lead to changes in functional, sensory, and nutritive values that reduce consumer acceptability of the product [4]. As known, the consumption of oxidized oil leads to a variety of diseases, such as atherosclerosis and cancer [10]. The level of oxidation of the containing potato chips oil, and the concomitant formation of off-flavors, is ultimately assessed by some tests such as the peroxide value (PV), acid value (as free fatty acids) (AV) and levels of conjugated dienes (CD) formed during oxidation [11]. Different chemical and natural antioxidants have been examined to retard the lipid oxidation of fats and oils in processed foods [4,12].

Probiotics are live microorganisms that, when administered in adequate amounts, confer health benefits to the host as defined by Food and Agriculture Organization/World Health Organization [13]. It was approved by Food and Drug Administration (FDA) as Generally Recognized as Safe (GRAS) [14]. Today, most commercially available probiotics belong to the genera *Lactobacillus* and *Bifidobacterium*. Many of them have already been used as probiotics in different dairy [15,16] and nondairy products [17,18]. Besides their innumerous gut health effects, they showed many benefits such as antimicrobial activity, ability to modulate the immune response, gastrointestinal ecosystems improvement [19,20] and anticancer activity [21]. Several in vitro studies have confirmed the strong antioxidant power of some probiotics, especially *Bifidobacterium longum* and *Lactobacillus helveticus* strains [10]. As examples, *Lactobacillus helveticus* in skim milk showed considerably strong antioxidant activity (62.32%) measured using O-phthaldialdehyde, 2, 2-diphenyl-1-picrylhydrazyl (DPPH) reagent [22]; bovine skim milk fermented by selected *L. helveticus* 474 strain showed high free radical (1,1-diphenyl-2-picrylhydrazyl) scavenging activity [23]. Also, whey protein isolates hydrolyzed by *Lactobacillus helveticus* T80 fermentation had strong antioxidant activity [24]. Encapsulated *Bifidobacterium longum* KACC 91,563 caused the least lipid oxidation level when used in sausage fermentation by Song et al. [25] in comparison with cell-free sausages. In addition, those two strains have beneficial antiobesity effects with different mechanisms, i.e., reduced lipid accumulation, lowering high-fat-diet-elevated lipopolysaccharide levels in blood and/or insulin resistance and hepatic steatosis formation. An excellent example demonstrating the potential of probiotics for fats reduction is represented by An and coworkers [26] who investigated the antiobesity and lipid-lowering effects of *Bifidobacterium longum* SPM 1205 and 1207 strains on high-fat-diet-induced obese rats. In addition, four *Bifidobacteria* (L75-4, M13-4 and FS3-1-1-2) strains showed a role in reducing serum and liver triglyceride and total cholesterol, as well as liver lipid deposition as reported by Yin et al. [27]. Plasma lipopolysaccharide was normalized to the control levels in the high-fat *Bifidobacterium longum*-treated rats [28]. A study by Arigoni et al. [29] also demonstrated the effect of *Lactobacillus helveticus* CNCM I-4095 strain in supporting weight management, which promoted weight loss by 15% after 40 days of administration and could be used to treat obesity.

Foods containing probiotic microorganisms are expected to have a promising future. The challenge is to maintain the viable cultures in the final product for the longest period. Freeze-drying (lyophilization or cryodesiccation) is the most commonly used technique for probiotic culture dehydration to stabilize probiotics in functional foods [30]. Freeze-drying involves the dehydration by sublimation of frozen ice present within the molecules. It is a preferred method for compounds that are thermally sensitive and prone to oxidation, since it operates under a high vacuum and at low temperatures [31]. Lyophilized probiotic bacteria have been extensively used for pharmaceutical applications [32] and foodstuffs such as sausages [33], soy bars [34], apple snacks [35] and many dairy products [36,37,38]. Chocolate, as the most consumed candy, although it is rich in fats and sugars, has also been tested as a carrier for lyophilized probiotics. For example, lyophilized *Lactobacillus casie* and *paracasie* maintained live at 10^6^–10^8^ CFU/g in milk chocolate for 1 year [39]. The viability of probiotic *Lactobacillus helveticus* bacteria (2.42 × 10^8^ CFU/g) in chocolate was achieved only up to 15 days of storage at 10 ± 2 °C [40], while *Lactobacillus plantarum* showed high stability in chocolate for 6 months [41]. However, the application of lactic acid bacteria and their physiological effect on potato chips has not yet been reported.

Given that potato chips are widely consumed around the world and the recent trend towards foods supplemented with live probiotics, this study was performed. Two probiotic lactic acid bacteria *Bifidobactrium longum* and *Lactobacillus helveticus* were chosen for the production of probiotic chips for their proved antioxidation and antiobesity effects [24,25,26,27,28,29]. First, this study aims to select the most appropriate protective media for probiotic lactic acid bacteria lyophilization. Second, it aims to investigate the effect of the lyophilized probiotic addition on the quality of potato chips and the shelf life of the stored product.

## 2. Materials and Methods

### 2.1. Raw Materials

Fresh potato (*Solanum toberosum* var. Cara) was purchased from the local market, Giza Governorate, Egypt. Its chemical composition is shown in Table 1. Refined sunflower oil was obtained from Arma Company for food products (10th Ramadan City, Egypt). It has the following profile (%): linoleic acid, 59; oleic acid, 30; stearic acid, 6; palmetic acid, 5 and vitamins A, D and H. Skim milk was purchased from Lamar Company for dairy products (Nubaria City, Egypt). All the used chemicals were fine analytical chemicals.

### 2.2. Potato Chips Preparation and Frying

Potatoes were washed, peeled, and then sliced into chips (1.2 mm thickness). For more crispness, they were soaked in CaCl_2_ (1%) for 10 min. A known amount (c. 2 kg) of refined sunflower oil was placed in a stainless steel pan fryer (50 cm diameter x 20 cm height) and heated at 180 ± 5 °C. The chips were deep oil fried for 5 min, drained to remove excess oil, then cooled [42].

### 2.3. Probiotic Lactic Acid Bacteria

Two probiotic lactic acid bacteria were used in this study. *Bifidobactrium longum* ATCC 15708 that was obtained from American Culture Collection, Manassas, VA, USA, and *Lactobacillus helveticus* LH-B02 (Chr. Hansen Laboratory Ireland Ltd., Little Island, Cork, Ireland). Stock cultures were stored at −20 °C in de Man, Rogosa and Sharpe (MRS) broth (Merck, Darmstadt, Germany) containing glycerol (20%). Working cultures were maintained anaerobically in MRS broth at 4 °C and were transferred to a new medium every month.

### 2.4. Probiotic Lactic Acid Bacteria Production

The lactic acid bacteria cells were prepared (before lyophilization) following procedures previously described [43] with minor adaptations. Two cryoprotective media were tested in this study. Skim milk (SM) medium contained 10% skim milk + 5% sucrose while, gelatin/glycerol (GG) medium (lactose free) contained 1.5% gelatin + 1% glycerol (from Sigma Aldrich Co.,Saint Louis, MO, USA). One hundred ml of fresh MRS was inoculated with 2.5 mL of maintained MRS broth culture and incubated anaerobically at 37 °C for 48 h in an Anaerobe Jar. Subsequently, the obtained inoculum was used to inoculate the bottle of MRS (containing 400 mL) and further incubated anaerobically at 37 °C for 24 h (stationary phase). Cells were harvested by centrifugation (Hermle, Z300, Gosheim, Germany) at 11,000× *g* for 5 min at 4 °C, then washed 3 times with sterilized distilled water. After this, the supernatant was discarded, and the harvested cells were resuspended (final viable counts in the range of 7–8 log CFU/mL) in 100 mL of SM medium. The same procedures were repeated, but the harvested cells were resuspended in 100 mL of GG medium. All the resuspended cells were frozen to −20 °C before the lyophilization.

### 2.5. Lyophilization and Survival Test

The frozen suspensions were dried-frozen (temperature −40 ± 2 °C; vacuum pressure 10^−1^ torr for 48 h) using a bench-top lyophilizer (Modulyo bench top freeze dryer, Edwards, Burgess Hill, UK). Viable cell counts were checked after freeze drying and in packed probiotic potato chips at different storage intervals (zero, 1, 2, 3 and 4 months) by the standard plate count method. For this, the dried powder or fried chips was aseptically rehydrated in sterile saline solution (NaCl, 0.85%) at room temperature for 10 min. One ml aliquots were serially diluted and plated; then, MRS agar medium was poured. After incubation for 2–3 days at 37 °C under anaerobic conditions, the colonies were counted and results were expressed as log CFU/g [44].

### 2.6. Probiotic Potato Chips Production and Storage Condition

Potato chips immediately after frying and cooling were pooled together and packaged in 20 × 10 cm polyethylene bags. Each bag was filled with chips (4.5 ± 0.2 g) and lyophilized lactic acid bacteria powder that sticks on the surface of potato chips in the final cell count (10^9^–10^10^ CFU/g), vacuumed and heat-sealed then stored at room temperature 30 ± 2 °C in a dark place for 4 months. Bags containing potato chips with no lactic acid bacteria were used as a control. Bags were removed from the storage carton monthly and the lactic acid bacteria viability was assessed as previously described.

### 2.7. Chemical Analysis

Chemical analysis of fresh potato (moisture, protein, fiber, fat and ash) was carried out according to A.O.A.C [45]. Total carbohydrates were determined by the Anthrone method [46] (after HCl hydrolysis, 2.5 N for 3 h), while reducing sugar content was determined by the 3, 5-Dinitrosalicylic acid test (after extraction by hot ethanol solution, 80%) [47].

After the storage period (the fourth month), the stored probiotic chips from 3 bags from each experiment were crushed and weighted. The oil content was determined using the Soxhlet apparatus. Lipid extraction was performed by chloroform/methanol mixture (1:1) as described by Petukhov et al. [42]. Peroxide value (PV) of extracted oil was determined according to Paquot [48] method II.D.13. Briefly, the sample is treated with a mixture of glacial acetic acid and chloroform then with a saturated potassium iodide solution. The liberated iodine is titrated with a standard solution of sodium thiosulfate (0.05 N) and expressed as meq O_2_/kg. Free fatty acid percent (acid value, AV) was determined as described by Atalay and Inanc [49] by titration with KOH (0.05 N) in the presence of phenolphthalein and expressed as Oleic acid. Each analysis was performed in triplicate.

### 2.8. Sensory Evaluation

An untrained panel of students and staff members (*n* = 20) at Food Science Department, Faculty of Agriculture, Cairo University evaluated the samples monthly for different sensory attributes such as taste, odor, color, appearance, texture and overall acceptance (using a 9-point hedonic scale). Samples were evaluated for their all sensory attributes where 9 and 1 represented liked extremely and disliked extremely, respectively [8].

### 2.9. Statistical Analysis

Experiments were conducted in triplicate and data were analyzed using the CoStat Microsoft program. Significant differences among means (*n* = 3) were determined by one-way ANOVA, using Duncan’s test at *p* < 0.05.

## 3. Results and Discussion

### 3.1. Survival of Probiotic Lactic Acid Bacteria

Probiotics, being live microorganisms, have a great difficulty being incorporated and still living at the time of food product consumption. Therefore, choosing the right strains, culture conditions, and cryoprotectants should be considered. Protective agents such as skim milk, whey, trehalose, glycerol, sucrose, and glucose are commonly employed to protect bacterial cultures [30]. To preserve lactic acid bacteria during the lyophilization process, two media (i.e., SM and GG) were evaluated as cryoprotectants. The cell viability before and after the lyophilization process are shown in Table 2. It can be observed that the GG medium was better for protecting *Bifidobacterium longum* after lyophilization, as 79.03% of its initial population was still live. On the other hand, *Lactobacillus helveticus* lost 66.66% of its viability after lyophilization in SM medium. GG medium had a significant negative effect in the decline of its viability by 92.28%. Lyophilization can cause many negative effects on the cells, such as a disruption of the cell walls resulting from the water vapor transportation to the surface of the sample for sublimation, collapse of protein and shrinkage [31]. Sharma et al. [50] reported skim milk as cryoprotectant for *Streptococcus thermophilus* strain. However, for *Lactobacillus* strains lyophilization, Yeo et al. [44] recommended a mix of 10% skim milk and 10% sucrose with 2.5% sodium glutamate.

Probiotic potato chips as a new probiotic product were assessed. The mean values of lyophilized probiotic cell viability in packed potato chips during storage for 4 months are represented in Table 3. Viability of lyophilized *Bifidobacterium longum* strain in SM medium gradually decreased (with a significant difference *p* < 0.05) but still maintained at 6.5 log CFU/g for 120 days. In contrast, in GG medium, departing from 10.14 log CFU/g, it decreased to 7.65 log CFU/g (99.67% loss) after 2 months of storage, then its viability was completely lost. With 10^6^–10^7^ CFU/g, potato chips containing *B. longum* lyophilized in SM medium are considered as a probiotic product because it is the standard limit accepted for probiotic products to be delivered to the consumers at the shelf-life end [16]. In the case of *Lactobacillus helveticus* strain, in SM medium, the viability decreased reaching 10^4^ CFU/g after the storage for four months. On the other side, it could not survive over one month in GG medium. It could be concluded that, under vacuum and high-oil content (potato chips) conditions, skim milk medium could protect both strains for 4 months compared to GG medium. That is supported by the previous results (Table 2), as GG medium caused the highest viability reduction of *L. helveticus* after the lyophilization step. The presence of milk solids is responsible of the difference of the two cryoprotectants during the storage with potato chips. The high nutrient content of milk might support their growth. Also, lactose, the principal sugar in milk, triggers the production of the inducible enzyme *β-*galactosidase, which sustains LAB growth [51]. In addition, cryoprotectants rich in amino acids and fermentable sugars (lactose and sucrose) can stabilize the lipid bilayer structure of the cell membrane in the absence of water [16]. *Bifidobacterium longum* viability in SM was higher than those reported by Nebesny et al. [52], who maintained lyophilized *Lactobacillus* in chocolate at 5 log CFU/g for 5 months storage at 30 °C. However, *Bifidobacterium thermophiles* NCIMB 702,554 maintained viability at 7.3 log CFU/g for 90 days at 25 °C [53]. While Guergoletto [25] maintained viable *Lactobacillus casei* in chocolate bars at 8.3 log CFU/g for 84 days at 25 °C, Mirkovic et al. [54] maintained encapsulated *L. plantarum* 299v in dark chocolate at 10^8^ CFU/g up to 6 months at 20 °C. In another probiotic product, soy protein bars, freeze-dried microcapsules of *Lactobacillus acidophilus* LA-2 remained in high number throughout 14 weeks at 4 °C [34]. In contrast, in probiotic *Lactobacillus plantarum*-enriched apple snacks dried by microwave-vacuum, the probiotic bacteria remained above 1 × 10^6^ CFU/g for 120 days at 25 °C [35]. Furthermore, freeze-dried immobilized *L. casei* ATCC 393 cells on casein were detected in yogurt at levels > 7 log CFU/g after cold storage for 28 days [38].

### 3.2. Chemical Evaluation of the Stored Potato Chips

**Figure 1:**
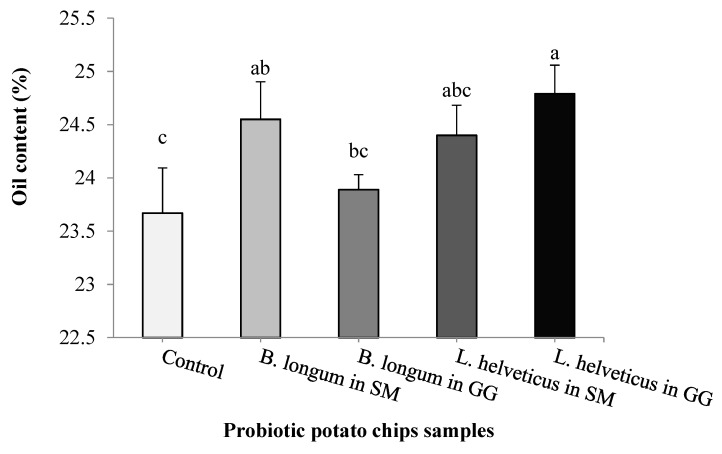
Oil content in different probiotic potato chips samples. Potato chips containing probiotic *Bifidobacterium longum* and *Lactobacillus helveticus* (lyophilized in skim milk or gelatin/glycerol medium) or without LAB (control), were analyzed after storage at 30 °C for 4 months. a–d Different letters in the columns differ signiﬁcantly (*p* < 0.05).

At the end of the storage time, the oil content of the different samples of probiotic potato chips and probiotic-free bags (control) was determined (Figure 1). The oil content ranged between 23.67% to 24.79%. Remarkably, there was no significant (*p* > 0.05) difference in the oil content values between probiotic products that contained both strains in all cryoprotective media. The significant difference was observed between *B. longum* (in SM medium), *L. helveticus* (in GG medium) with control bags, which is not biologically meaningful (1–1.12%).

**Figure 2:**
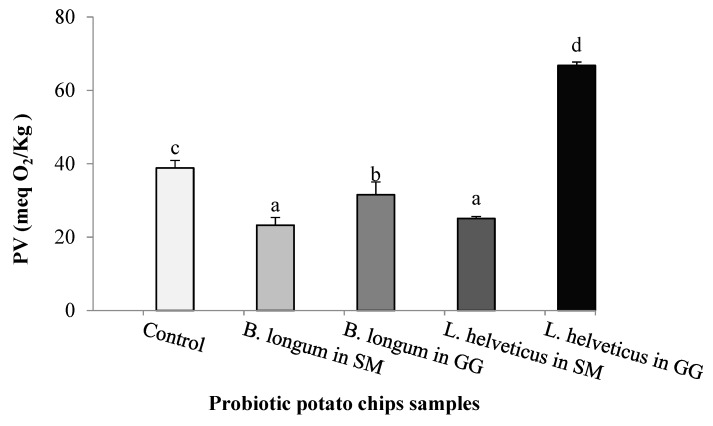
Peroxide value (PV) of the extracted oil from different probiotic potato chips samples. Potato chips containing probiotic *Bifidobacterium longum* and *Lactobacillus helveticus* (lyophilized in skim milk or gelatin/glycerol medium) or without LAB (control), were analyzed after storage at 30 °C for 4 months. a–d Different letters in the columns differ signiﬁcantly (*p* < 0.05).

The primary products of lipid oxidation are hydroperoxides. Therefore, PV is an importance index to assess the level of lipid oxidation in products containing oils during storage. It reflects the amount of hydro peroxides and secondary oxidation products (ketones and aldehydes) in fat [55]. Peroxide value (PV) of the extracted oil was determined in all samples and represented in Figure 2. Although there were no significant differences in oil content of different probiotic chip samples (Figure 1), the probiotic LAB strain and the lyophilization medium significantly affected the PV values. Interestingly, PV was significantly reduced by 40.13% and 35.47% in bags containing *Bifidobacterium longum* and *Lactobacillus helveticus* lyophilized in SM medium, respectively, compared to the control bags (38.85 meq O_2_/kg). In GG medium, lyophilized *B. longum* strain decreased PV by 19%. This could be explained as this strain remained alive for 4 months in an adequate population. In contrast, the highest PV value was recorded by *Lactobacillus helveticus* in GG medium (1.71 times higher than the control chips). The explanation of that high increment could be explained as *L. helveticus* is hydrogen peroxide-producing strain [56]. It may be highly producing H_2_O_2_ during storage in GG medium. Hydrogen peroxide is known as an oxidizer [57] and can promote the oxidation of potato chips’ oil. On the other side, the antioxidant activity of *Bifidobacterium longum* to inhibit lipid oxidation was proven by many researchers [58,59]. They explained that activity by the fact that the *Bifidobacterium longum* strain may secrete an extensive amount of polyphenolic and phenolic compounds that minimized lipid oxidation. In an earlier study, PV ranged from 0.12 to 7.4 meq O_2_/kg fat during two months storage of fried potato crisps at room temperature, which was recorded by Abong et al. [9]. Rababah et al. [12] using grape seed extracts at 1000 ppm minimized the peroxide value development in potato chips during 90 days of storage.

**Figure 3:**
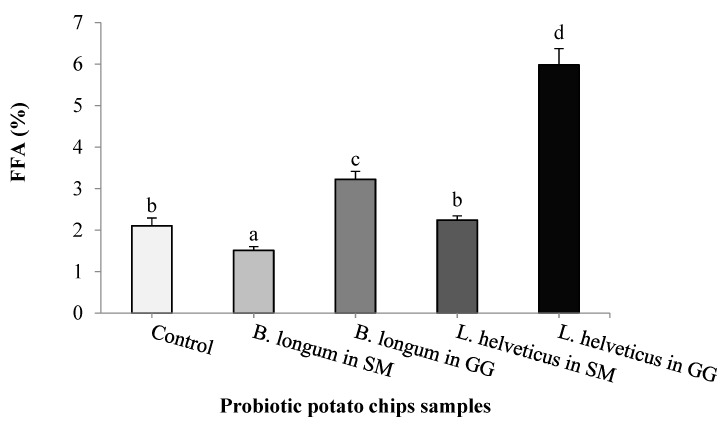
Acid value (AV) as free fatty acid (FFA) percent of the extracted oil from different probiotic potato chips samples. Potato chips containing probiotic *Bifidobacterium longum* and *Lactobacillus helveticus* (lyophilized in skim milk or gelatin/glycerol medium) or without LAB (control), were analyzed after storage at 30 °C for 4 months. a–d Different letters in the columns differ signiﬁcantly (*p* < 0.05).

Acid value (AV) indicates the amount of free fatty acids in the food products and is a sign of oil stability during storage. High AV may cause diarrhea, gastrointestinal discomfort, and even liver damage [60]. In the present study, results in Figure 3 revealed that by lyophilization in SM media, *B. longum* was the most effective in minimizing free fatty acid formation in potato chips, followed by *L. helveticus*. Gelatin/glycerol (GG) medium has a negative effect on AV, as chips containing *B. longum* and *L. helveticus* formed the most significant FFA percent values (3.22%, 5.98%, respectively) in potato chip bags. Abong et al. [9] reported a significant increase in free fatty acid content in fried potato crisps during storage at room temperature (25–30 °C) for a total period of 24 weeks. No previous research was conducted to evaluate probiotic lactic acid bacteria in minimizing the peroxide or acid value in potato chips.

### 3.3. Sensory Evaluation of the Stored Potato Chips

**Figure 4:**
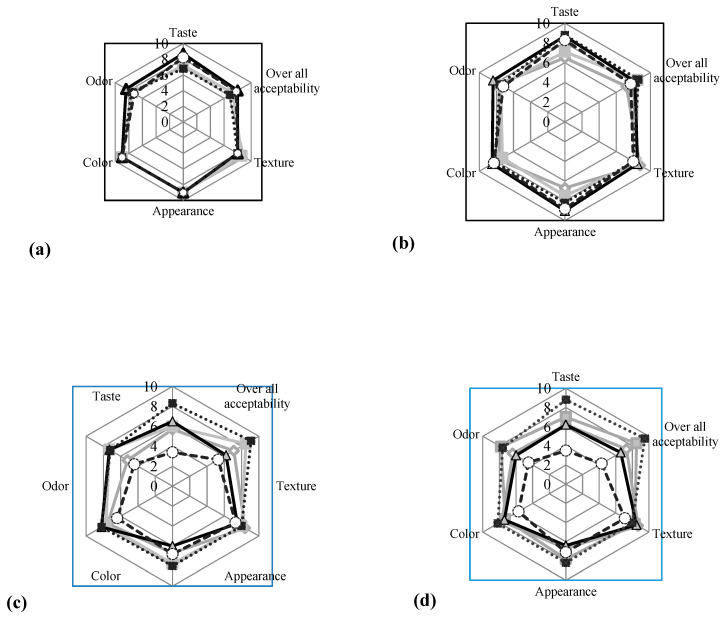

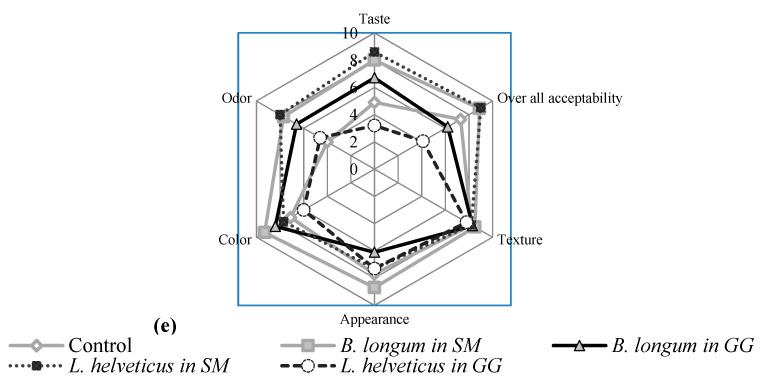
Sensory evaluation of probiotic potato chips during storage for 4 months at 30 °C. (**a**) At zero time, (**b**) After storage for one month, (**c**) After storage for two months, (**d**) After storage for three months and (**e**) After storage for four months.

Deep frying of potato chips gives them a unique appearance, flavors and texture, leading to a highly palatable product [55]. The mean values of the sensory characteristics of different potato samples are shown in Figure 4. Probiotic potato chips products were found acceptable by the consumers. By increasing the storage time, the sensory evaluation showed significant differences (*p* < 0.05) between the analyzed samples. Although the packed potato chips containing *B. longum* and *L. helveticus* in GG medium had the highest taste and overall acceptability scores at the experiment commencement, it declined to the lowest scores after the fourth month of storage, especially in bags containing *L. helveticus* in GG medium (overall acceptability score of 4.1). Overall, chips containing *B. longum* and *L. helveticus* in SM medium had the highest scores for taste, odor and overall acceptability for the next 3 months compared to chips without LAB (control). The greatest overall acceptability, taste and odor scored by *B. longum* in SM medium may be correlated to its viability and low PV and AV scores after 4 months of storage. In contrast, the decrease in sensory scores in bags containing *B. longum* and *L. helveticus* in GG medium may be related to the off-flavors released during storage. This is probably due to the formed free fatty acids and peroxides in the absence of viable probiotic cells. That means it is technically possible to incorporate *B. longum* into potato chips bags to preserve its quality during the shelf life.

## 4. Conclusions

Two cryoprotective media were compared to evaluate their suitability for probiotic lactic acid lyophilization and its protective effect in potato chips. GG and SM media were the most suitable for protecting *Bifidobacterium longum* and *Lactobacillus helveticus*, respectively. On the other side, potato chips proved a suitable vehicle for probiotics. Skim milk medium seemed to be the most useful medium in probiotic potato chip production, due to high recorded viability maintenance. Among the tested probiotics, *Bifidobacterium longum* was the best strain, as it preserved its viability at 10^6^ CFU/g for 4 months, and decreased the PV and AV of oil in potato chips. As this strain could limit excessive amounts of reactive radicals in vivo, it may contribute to prevent and control several diseases associated with oxidative stress and over-consumption of potato chips. Potato chips containing *B. longum* have promising characteristics, with improved stability and sensory characteristics to meet the preferences and demands of consumers. In conclusion, we recommend consuming such invented product in moderation to benefit from the probiotics with their antiobesity influence without impairment to human body.

## Figures and Tables

**Table 1:** Chemical analysis of potato *Solanum toberosum* var. Cara.

Component	Mean (%) ± SD
Moisture	73.83 ± 0.83
Protein	5.75 ± 0.02
Fat	1.24 ± 0.06
Total carbohydrate	13.88 ± 0.15
Reducing sugar	0.27 ± 0.02
Crude fiber	2.70 ± 0.02
Ash	2.85 ± 0.87

**Table 2:** Viability of probiotic lactic acid bacteria (LAB) as affected by lyophilization (means ± standard deviation (SD)).

Lactic Acid Bacteria	Cryo-Protective Medium *	Cell Viability (Log CFU/g)		Viability Loss (%)
		Before Lyophilization	After Lyophilization	
*B. longum*	SM	9.22 ± 0.016	8.96 ± 0.033	44.68 ^c^
	GG	9.21 ± 0.014	9.11 ± 0.047	20.97 ^a^
*L. helveticus*	SM	9.02 ± 0.029	8.82 ± 0.060	36.66 ^b^
	GG	9.01 ± 0.020	7.91 ± 0.007	92.28 ^d^

* SM: 10% skim milk + 5% sucrose, or GG: 1.5% gelatin + 1% glycerol. ^a–d^ Means within rows with different superscripts differ signiﬁcantly (*p* < 0.05).

**Table 3:** Viability of lyophilized probiotic lactic acid bacteria (LAB) in the stored potato chips.

Potato Chips Containing LAB Samples	Time (Month)				
	0	1	2	3	4
	Cell Viability (Log CFU/g)				
*B. longum* in SM ^a^	* 10.20 ^bA^ ± 0.03	10.15 ^aB^ ± 0.00	9.55 ^aC^ ± 0.07	7.81 ^aD^ ± 0.02	6.50 ^aE^ ± 0.04
*B. longum* in GG	10.14 ^bA^ ± 0.04	9.22 ^bB^ ± 0.03	7.65 ^bC^ ± 0.01	0.00 ^cD^ ± 0.00	0.00 ^cD^ ± 0.00
*L. helveticus* in SM ^b^	10.63 ^aA^ ± 0.05	7.11 ^cB^ ± 0.02	4.28 ^cC^ ± 0.00	4.28 ^bD^ ± 0.02	4.06 ^bE^ ± 0.08
*L. helveticus* in GG	9.90 ^cA^ ± 0.07	4.57 ^dB^ ± 0.03	0.00 ^dC^ ± 0.00	0.00 ^dC^ ± 0.00	0.00 ^cC^ ± 0.00

* The experimental values (means ± SD) followed by different small letters a–d (within rows) and capital letters A–D (within columns) are significantly different (*p* < 0.05). ^a^ *Bifidobacterium longum* lyophilized in skim milk (SM) or gelatin/glycerol (GG) medium. ^b^ *Lactobacillus helveticus* lyophilized in skim milk (SM) or gelatin/glycerol (GG) medium.
